# Preoperative chemotherapy with docetaxel, cisplatin, and 5-fluorouracil for locally advanced esophageal carcinosarcoma: a case report and review of the literature

**DOI:** 10.1186/s40792-018-0425-4

**Published:** 2018-02-17

**Authors:** Tomoko Yoshimoto, Shinichiro Kobayashi, Kengo Kanetaka, Kazuma Kobayashi, Yasuhiro Nagata, Michi Morita, Yuriko Isagawa, Naoe Kinoshita, Mitsuhisa Takatsuki, Susumu Eguchi

**Affiliations:** 1 Department of Surgery, Nagasaki University Graduate School of Biomedical Sciences, Sakamoto 1-7-1, Nagasaki, 852-8102 Japan; 2 Center for Comprehensive Community Care Education, Nagasaki University Graduate School of Biomedical Sciences, Sakamoto 1-12-4, Nagasaki, 852-8523 Japan; 3 Department of Pathology, Saiseikai Nagasaki Hospital, Katafuchi 2-5-1, Nagasaki, 850-0003 Japan

**Keywords:** Docetaxel, Cisplatin, 5-Fluorouracil, Esophagus, Carcinosarcoma

## Abstract

**Background:**

Esophageal carcinosarcoma is a relatively rare malignant neoplasm composed of both epithelial carcinomatous and mesenchymal sarcomatous elements. There is no recommended clinical treatment for esophageal carcinosarcoma because of the rarity of the disease. This report describes a case of esophageal carcinosarcoma that was effectively treated with docetaxel, cisplatin, and 5-fluorouracil as preoperative chemotherapy.

**Case presentation:**

A 73-year-old man had a chief complaint of dysphagia with epigastric pain. Esophagogastroduodenoscopy (EGD) revealed a polypoid neoplasm combined with an infiltrative ulcer that exhibited a mixture of squamous cell carcinoma and spindle cell sarcoma histologically. Computed tomography findings showed swollen lymph nodes in the mediastinum and around the cardia. We diagnosed esophageal carcinosarcoma cT3N1M0 cStage III. After preoperative chemotherapy with docetaxel, cisplatin, and 5-fluorouracil, the patient underwent thoracoscopic esophagectomy with three-field lymph node dissection. Histological findings revealed that the sarcomatous component had completely disappeared and the carcinomatous component was only confined by the basement membrane with scar formation of the muscularis propria. Mural fibrotic lesions were observed in several resected regional lymph nodes. Hence, immediately after preoperative therapy, the esophageal carcinosarcoma was diagnosed as ypTisN0M0 fStage I. The patient remained alive without tumor recurrence at 12 months after the operation.

**Conclusions:**

A review of the literature revealed that there is still no established therapeutic strategy for locally advanced esophageal carcinosarcoma, especially against the sarcomatous component. We herein provide the first report in which the sarcomatous component showed a complete response to preoperative chemotherapy with docetaxel, cisplatin, and 5-fluorouracil. Preoperative chemotherapy with docetaxel, cisplatin, and 5-fluorouracil followed by esophagectomy with extended lymphadenectomy may achieve definitive treatment for locally advanced esophageal carcinosarcoma.

## Background

Esophageal carcinosarcoma is a relatively rare malignant neoplasm composed of both epithelial carcinomatous elements and mesenchymal sarcomatous elements [1, 2]. Although esophageal carcinosarcoma has a different cellular pleomorphism, the sarcomatous elements are considered to have a monoclonal epithelial origin with sarcomatoid differentiation. Because of the rarity of the disease and lack of evidence regarding the response of the sarcomatous element, no robust conclusion has been reached about whether perioperative therapy can improve the control of locally advanced esophageal carcinosarcoma. We herein report a case of locally advanced esophageal carcinosarcoma treated with docetaxel, cisplatin, and 5-fluorouracil (DCF) as preoperative chemotherapy.

## Case presentation

**Fig. 1:**
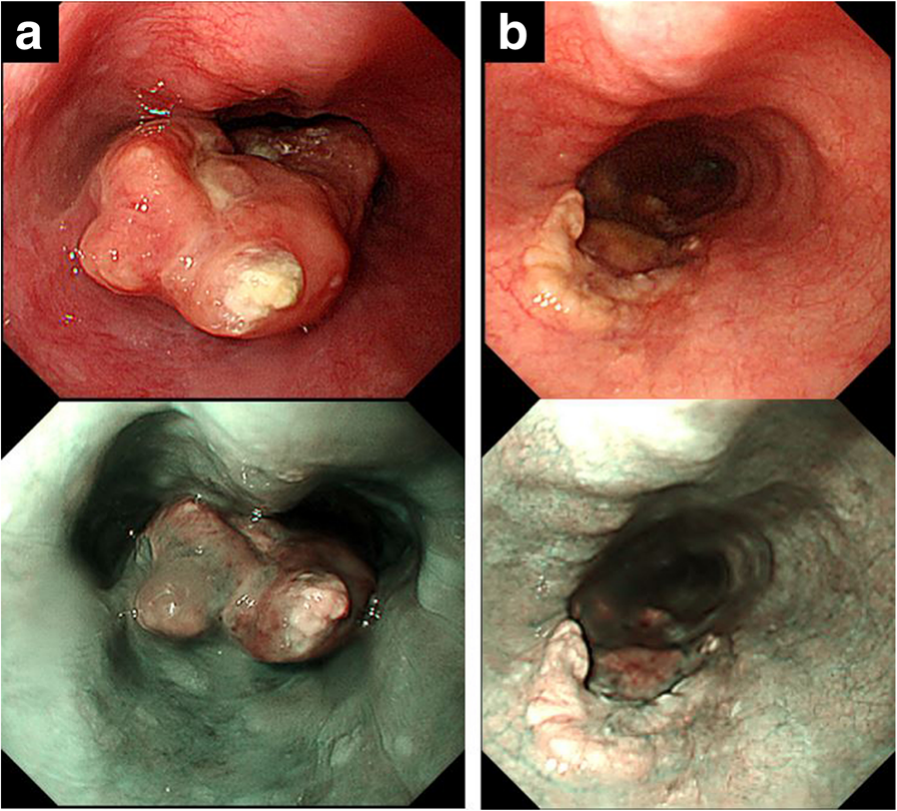
Endoscopic findings. **a** A protruding tumor was observed in the middle of the thoracic esophagus. **b** After two courses of preoperative chemotherapy, the tumor had reduced in size

**Fig. 2:**
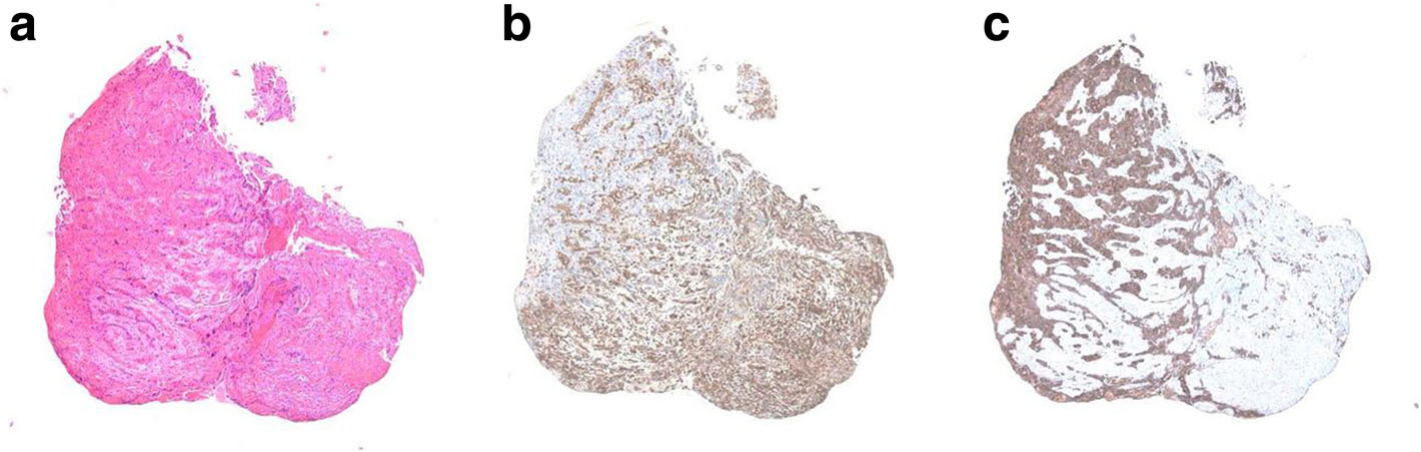
Microscopic and immunohistochemical findings of a biopsy specimen from the tumor. **a** The histological findings of the biopsy specimen showed squamous cell carcinoma with spindle cell components. **b**, **c** Immunohistochemical evaluation of the biopsy specimen revealed that the squamous cell carcinoma cells were positive for pan-cytokeratin, while the spindle cells were positive for vimentin. **a** Hematoxylin and eosin, **b** vimentin, and **c** pan-cytokeratin (× 40)

**Fig. 3:**
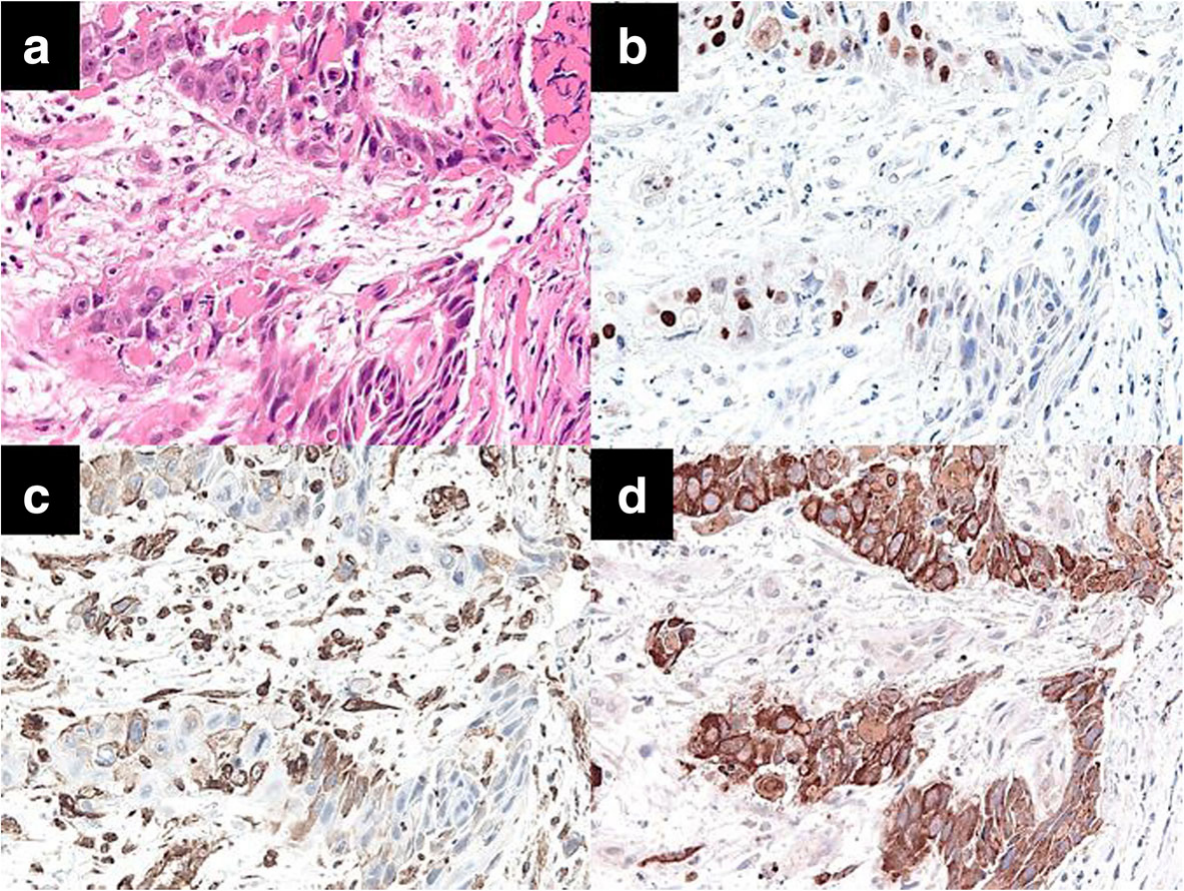
Microscopic and immunohistochemical findings of a biopsy specimen from the transitional zone between the carcinomatous and sarcomatous components. **a**–**d** In the transitional zone between the two components, there were scattered malignant cells expressing pan-cytokeratin, vimentin, or p63. **a** Hematoxylin and eosin, **b** p63, **c** vimentin, and **d** pan-cytokeratin (× 200)

**Fig. 4:**
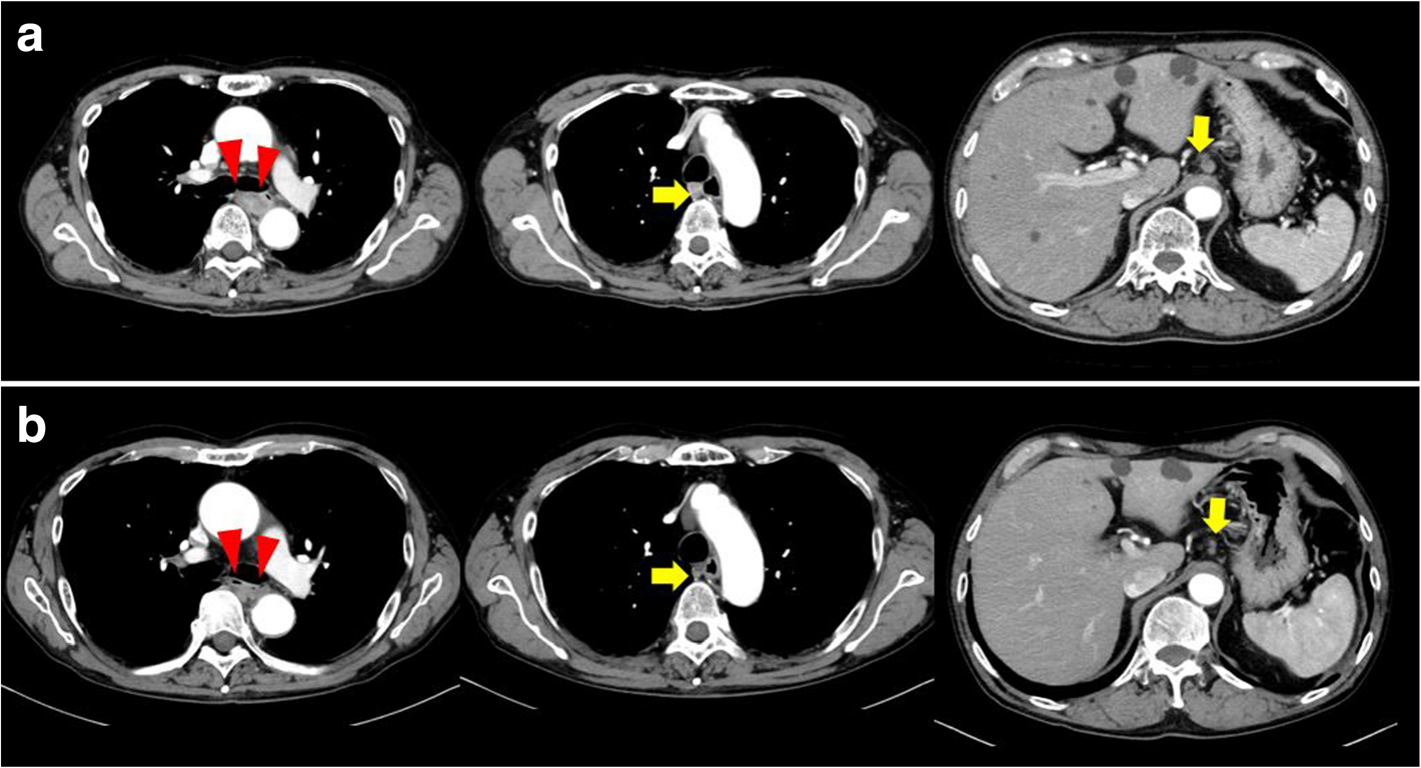
Computed tomography findings. **a** Before preoperative chemotherapy, a tumor was observed in the middle of the esophagus (red arrows) with swollen lymph nodes in the mediastinum and around the cardia (yellow arrows), but there was no distant metastasis. **b** After preoperative chemotherapy, the tumor (red arrows) and swollen lymph nodes in the mediastinum and around the cardia (yellow arrows) were reduced

**Fig. 5:**
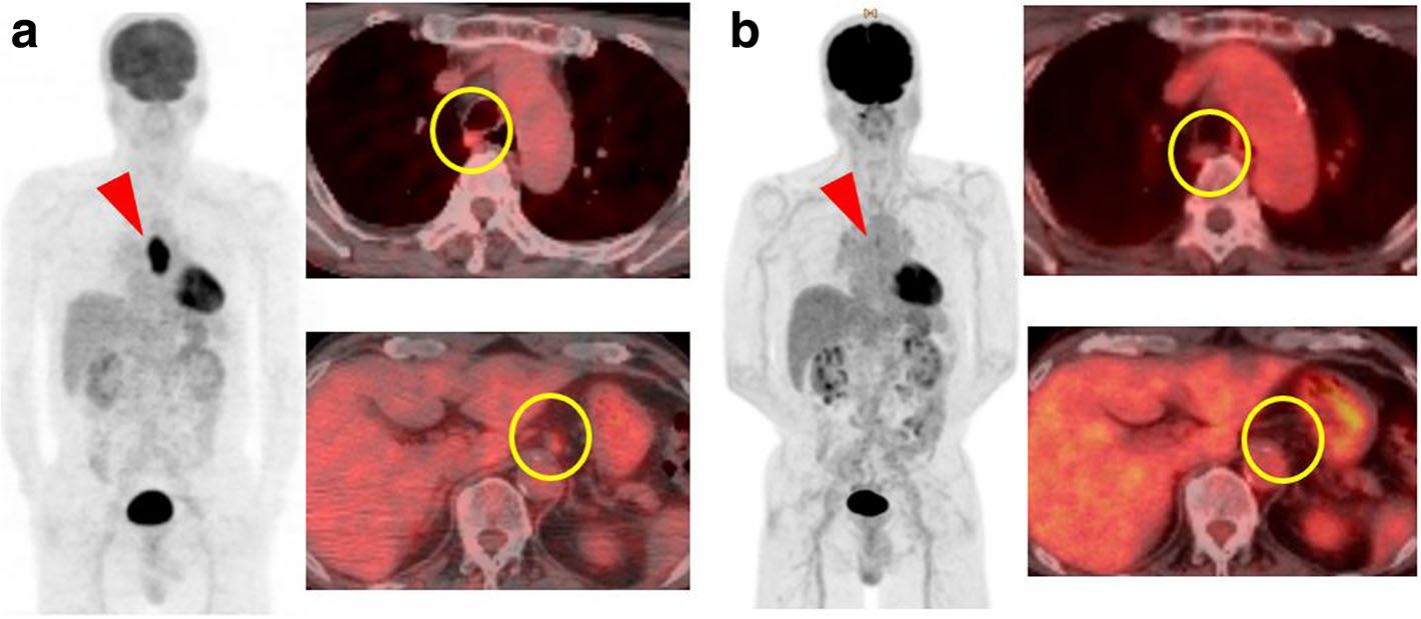
Fluorodeoxyglucose-positron emission tomography (FDG-PET) findings. **a** Before preoperative chemotherapy, there was high FDG uptake by the tumor in the esophagus (a red arrow) and the swollen lymph nodes in the mediastinum and around the cardia (yellow circles). **b** After preoperative chemotherapy, the tumor had almost disappeared (a red arrow) and the swollen lymph nodes in the mediastinum and around the cardia had decreased (yellow circles)

**Fig. 6:**
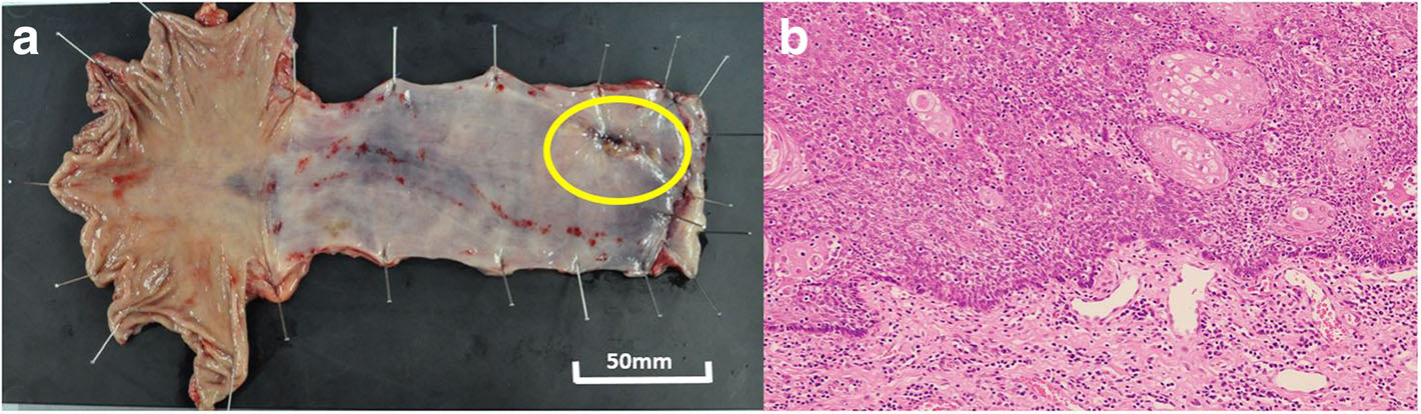
Macroscopic and microscopic findings of the resected specimen. **a** The scar without an obvious neoplasm measured 40 mm (bar 50 mm). **b** A histopathological examination demonstrated that only the carcinomatous component was present in situ and the sarcomatous component had disappeared (× 200)

A 73-year-old man was admitted to a clinic with epigastric pain. His family history was unremarkable, and he took medication for hypertension. When he was referred to our hospital for further examination, his epigastric pain had improved and his physical findings were normal. All laboratory data and serum tumor markers were within the normal limits. Esophagogastroduodenoscopy (EGD) revealed a large polypoid neoplasm combined with an infiltrative ulcer, 6 cm in diameter, in the thoracic esophagus (Fig. 1a). The histological findings of a biopsy specimen from the tumor showed squamous cell carcinoma with spindle cell components. Immunohistochemical evaluation of the biopsy specimen revealed that the squamous cell carcinoma cells were positive for pan-cytokeratin and p63, while the spindle cells were positive for vimentin (Fig. 2). Transitional features between the carcinomatous and sarcomatous components were evident (Fig. 3). Therefore, the tumor was diagnosed as carcinosarcoma. A computed tomography (CT) examination revealed that the neoplasm expanded into the thoracic esophagus without invasion to the adjacent organs and that swollen lymph nodes were present in the mediastinum and around the cardia (Fig. 4a). No metastatic regions were found in distant organs, including the lungs and liver. Fluorodeoxyglucose-positron emission tomography (FDG-PET) showed high FDG uptake by the neoplasm in the esophagus and swollen lymph nodes in the mediastinum without involvement of distant lymph nodes and organs (Fig. 5a). The clinical diagnosis according to the eighth edition of the Union for International Cancer Control (UICC) was T3N1M0 Stage III esophageal cancer. Preoperative DCF chemotherapy was proposed. The DCF therapy, which consisted of intravenous docetaxel (60 mg/m^2^, day 1), cisplatin (60 mg/m^2^, day 1), and continuous 5-fluorouracil (800 mg/m^2^, days 1–5), was administered twice at an interval of 4 weeks [3]. After two courses of DCF therapy, the neoplasm was extremely reduced on EGD (Fig. 1b), and FDG-PET and CT examinations showed no FDG uptake by the tumor and lymph nodes (Figs. 4b and 5b). Severe neutropenia and stomatitis developed during the second course of DCF therapy. The patient underwent thoracoscopic esophagectomy with three-field lymph node dissection after the preoperative chemotherapy. The resected specimen revealed a scar with no obvious neoplasm (Fig. 6a). The histological findings revealed a nodular scar, 4 cm in diameter, within the muscularis propria that seemed to be a degenerated tumor. The sarcomatous element had completely disappeared, and the carcinomatous element was only observed in situ (Fig. 6b). Several scars were observed in the resected regional lymph nodes. Hence, immediately after preoperative therapy, the esophageal carcinosarcoma was diagnosed as ypTisN0M0 Stage I according to the eighth edition of the UICC. He was discharged on postoperative day 17 with no complications. He remained disease-free for 12 months after the surgery.

### Discussion

Esophageal carcinosarcoma is a rare malignant neoplasm that accounts for 0.1 to 2.8% of all esophageal malignant tumors [1]. In general, esophageal carcinosarcoma tends to present as a bulky intraluminal polypoid mass and to occur more commonly in men, typically those aged 60 to 70 years. About 60% of tumors arise in the mid-esophagus, with nearly one third in the distal esophagus [4]. The prognosis of esophageal carcinosarcoma remains unclear because of the rarity of the disease. Esophageal carcinosarcoma may have a better prognosis than esophageal squamous cell carcinoma because the early onset of symptoms caused by intraluminal growth allows earlier detection of the disease [1, 2, 4]. Meanwhile, some investigators have reported a high frequency of distant metastasis and poorer long-term prognosis [5–7]. Kuo et al. [7] reported that the 1- and 2-year survival rates in 12 patients with esophageal carcinosarcoma were 50 and 25%, respectively. Other systematic reviews have shown that patients with T1 esophageal carcinosarcoma had a higher rate of lymph node metastasis and a lower 5-year survival rate than patients with T1 esophageal squamous cell carcinoma [5, 6]. Iyomasa et al. [8] reported that patients with esophageal carcinosarcoma sometimes developed late recurrence in the form of hematogenous metastasis after surgery. Thus, aggressive treatments are required for not only the carcinomatous component but also the sarcomatous component in locally advanced esophageal carcinosarcoma.

Esophageal carcinosarcoma is defined as a neoplasm with both epithelial carcinomatous and sarcomatous components [2]. Immunohistochemical analysis is necessary for the diagnosis of carcinosarcoma. Immunohistochemically, epithelial membrane antigen, carcinoembryonic antigen, cytokeratin, and synaptophysin are highly specific markers for carcinomatous elements, while vimentin, smooth muscle antigen, and desmin are specific for sarcomatous elements [6, 9]. Although the pathogenesis of the sarcomatous component in esophageal carcinosarcoma remains unclear, the sarcomatous component may arise from epithelial–mesenchymal transition (EMT) of the carcinomatous component [10, 11]. In carcinosarcoma, tumor cells are expected to lose their epithelial phenotype and gradually and sequentially acquire a mesenchymal phenotype during the EMT process [10]. In the present case, the biopsy specimen from the tumor was composed of both carcinomatous and sarcomatous elements. The carcinomatous component was positive for cytokeratin, while the sarcomatous component was positive for vimentin. A transitional zone was observed between the two components. The transitional zone consisted of scattered carcinoma cells expressing vimentin or p63, a marker of progenitor cells associated with inhibition of EMT-positive cells in the transitional zone [12]. In the present case, all malignant cells in the carcinomatous element were positive for p63, while almost all malignant cells in the sarcomatous element were negative for p63. The biological features of the transitional zone suggested that the sarcomatous element may have originated from the carcinomatous element via EMT.

There is no recommended clinical treatment for esophageal carcinosarcoma because of the rarity of the disease. Esophageal carcinomas have been treated with the multidisciplinary approach of surgery, chemotherapy, and radiotherapy. In Japan, preoperative chemotherapy with cisplatin and 5-fluorouracil followed by esophagectomy and regional lymph node resection is the standard treatment for locally advanced esophageal squamous cell carcinoma [13]. In addition, preoperative DCF chemotherapy has been reported to be safe and tolerable in patients with locally advanced esophageal cancer [3].

Docetaxel is an anticancer agent used to treat sarcomas in several fields [14]. The efficacy of docetaxel for bone and soft tissue sarcomas has been proven, mainly in combination regimens with gemcitabine [15–17]. Investigators in the gynecological field reported that leiomyosarcoma and endometrial stromal sarcoma treated with docetaxel-based chemotherapy showed favorable response rates [18–20]. Thus, we selected DCF therapy as preoperative therapy in the present case. In fact, the endoscopic findings revealed that the tumor dramatically decreased in size, although the clinical therapeutic effect of DCF therapy was stable disease according to the Response Evaluation Criteria in Solid Tumors guideline [21]. The postoperative pathological examination showed the carcinomatous element was slightly retained, but no sarcomatous element was observed. Therefore, the patient was classified as having a near pathological complete response, defined as 1 to 10% residual tumor cells with no tumor remaining in the resected lymph nodes [22]. DCF therapy would be effective for the carcinomatous element, while docetaxel may also be effective for the sarcomatous element.

We searched for articles in PubMed published since 1990 containing the key terms “esophagus” and “carcinosarcoma.” We identified seven cases of esophageal carcinosarcoma treated with preoperative therapy [7, 23–25]. Table 1 shows the details of these cases as well as our case. Six patients underwent chemoradiotherapy, and only one patient was treated with chemotherapy. A clinical response to preoperative therapy was observed in six patients (three patients with a partial response and three patients with stable disease). A pathological response to preoperative therapy was observed in four patients, including three patients treated with chemoradiotherapy or DCF therapy who obtained a near pathological complete response. Meanwhile, one patient treated with 5-fluorouracil and cisplatin obtained an insufficient pathological response to preoperative therapy. Thus, chemoradiotherapy or DCF therapy may be accepted as preoperative therapy for downstaging of locally advanced esophageal carcinosarcoma.

**Table 1:** Esophageal carcinosarcoma treated with preoperative therapy

First author	Year	Age	Sex	Stage	Preoperative therapy	Clinical response	Pathological response	Prognosis
Zuiki [23]	2009	50	Male	II	CRT (FP+62 Gy)	PR	ND	36 months alive
Zuiki [23]	2009	66	Male	I	CRT (FP+40.2 Gy)	PR	ND	19 months alive
Kobayashi [24]	2010	68	Male	III	CRT (S-1/CDDP+40 Gy)	PR	npCR	60 months alive
Kobayashi [24]	2010	64	Male	III	CRT (FP+38 Gy)	SD	npCR	11 months dead
Kuo [7]	2010	68	Male	III	CRT (ND)	ND	ND	27 months alive
Kuo [7]	2010	45	Male	IV	CRT (ND)	ND	ND	6 months alive
Kobayashi [25]	2015	69	Male	II	FP	SD	NC	60 months alive
Present case	2016	73	Male	III	DCF	SD	npCR	12 months alive

*CRT* chemoradiotherapy, *PR* partial response, *npCR* near pathological complete response, *SD* stable disease, *FP* 5-fluorouracil and cisplatin, *S-1/CDDP* S-1 and cisplatin, *NC* no change, *DCF* docetaxel, cisplatin, and 5-fluorouracil, *ND* not described

FDG-PET scans are well established for determining the stage, prognosis, and efficacy of preoperative therapy in squamous cell carcinoma [26]. Moreover, the standardized uptake values of carcinosarcoma are higher than those of squamous cell carcinoma regardless of differentiation type because the sarcomatous element of carcinosarcoma shows higher FDG uptake than the carcinomatous element [27, 28]. In the present case, the sarcomatous element completely disappeared after preoperative chemotherapy in the pathological evaluation as shown by FDG-PET scans, which showed no FDG uptake by the tumor and lymph nodes. Thus, FDG-PET scans can be a useful tool for predicting the therapeutic response and surveillance of esophageal carcinosarcoma.

## Conclusions

We have described a case of esophageal carcinosarcoma that was effectively treated with preoperative DCF chemotherapy. Preoperative chemotherapy with DCF may be an option for locally advanced esophageal carcinosarcoma.

## Abbreviations

CT: Computed tomography
DCF: Docetaxel, cisplatin, and 5-fluorouracil
EGD: Esophagogastroduodenoscopy
EMT: Epithelial–mesenchymal transition
FDG-PET: Fluorodeoxyglucose-positron emission tomography
UICC: Union for International Cancer Control
